# Promoting Immune Response of Human Vascular Endothelial Cells by Bevacizumab: Insights into the Immune Supportive Role of Anti-VEGF Therapy

**DOI:** 10.3390/ijms26136280

**Published:** 2025-06-29

**Authors:** Haiyan Jia, Anna Nowocin, Chris Burns, Meenu Wadhwa

**Affiliations:** Biotherapeutics and Advanced Therapies, Science and Research Group, Medicines and Healthcare Products Regulatory Agency, Blanche Lane, South Mimms, Potters Bar, Hertfordshire EN6 3QG, UK; anna.nowocin@mhra.gov.uk (A.N.); chris.burns@mhra.gov.uk (C.B.); meenu.wadhwa@mhra.gov.uk (M.W.)

**Keywords:** immunotherapy, immunosuppression, anti-VEGF, Bevacizumab, endothelial cells, T cells

## Abstract

Compelling clinical evidence strongly indicates that anti-angiogenesis therapeutics including Bevacizumab, a humanised anti-VEGF mAb, can alleviate the resistance to immunotherapy. We explored the direct modulation of Bevacizumab on endothelial cell (EC) immune response including surface expression of adhesion and MHC molecules and EC-elicited proliferation of immune cells under inflammatory conditions. Flow cytometry showed that addition of VEGF inhibited TNF-α stimulation of expression of ICAM-1 and VCAM-1 on HUVECs, whereas Bevacizumab enhanced this TNF-α-stimulated expression. The presence of MHC Class I on HUVECs was decreased by VEGF and increased by TNF-α, respectively. Bevacizumab reversed VEGF downregulation and promoted TNF-α upregulation of MHC class I expression, suggesting that anti-VEGF treatment can boost the endothelial immunological reaction, a prerequisite for immune cell trafficking. Functionally, real-time monitoring of the proliferation of human PBMCs co-cultured on HUVEC monolayers over 3 days showed opposing effects on the proliferation of PBMCs between VEGF and TNF-α. Consistently, Bevacizumab antagonised VEGF suppression and sensitized TNF-α activation of PBMC growth over the time course. In line with these findings, Bevacizumab increased the surface expression of CD69 on VEGF-treated T cells collected from PBMCs after 3-day co-cultures with HUVECs. Furthermore, the proliferation of CD3+, CD8+ and CD4+ T cells was promoted via Bevacizumab. Collectively, this study demonstrates that targeting VEGF can enhance the immune response of ECs required for T cell recruitment. Our findings provide insights to a deeper understanding of increased vascular inflammatory response conferred by anti-VEGF treatment in addition to inhibiting angiogenesis, which supports its favourable dual role in the positive immunological synergism with immunotherapy.

## 1. Introduction

The understanding of tumour-induced immune tolerance has led to the development of innovative treatments based on inhibition of negative immune regulation through targeting immune checkpoint molecules, including cytotoxic T lymphocyte antigen 4 (CTLA-4), programmed cell death protein 1 (PD-1) and programmed cell death ligand 1 (PD-L1) [1]. This immune checkpoint therapy to boost natural immunity against malignancy has revolutionised the treatment of advanced cancers such as metastatic melanoma, renal cell carcinoma and non-small-cell lung cancer, significantly prolonging patient survival [2]. However, durable clinical responses to most cancers are limited to a minority of patients, indicating the existence of additional tumour immunosuppressive mechanisms to mediate immune escape. Multiple mechanisms of resistance to immune checkpoint inhibitors have been hypothesised. Inadequate CD8+ cytotoxic T lymphocyte infiltration into the tumour microenvironment (TME) alongside the immunosuppressive effects of other TME residential immune and stomal cells is believed to play a pivotal role in cancer progression and therapeutic response [3,4,5,6].

Vascular endothelial growth factor (VEGF) is a vital player in the TME, which through its VEGF receptor 2 (VEGFR2) exhibits a dual role in regulating the functionality of vascular endothelial cells (ECs) and immune cells, the two major components of the TME [7]. Besides promoting tumour angiogenesis through the stimulation of EC migration and proliferation, VEGF produced by tumour cells can modulate the function of CD8+ cytotoxic T lymphocytes and immune suppressive cells in the TME, inducing tumour-associated immunosuppression [8]. VEGF directly inhibits the development, trafficking and recruitment of CD8+ cytotoxic T lymphocytes and the maturation of dendritic cells [9,10,11,12,13] and promotes the proliferation and TME infiltration of immunosuppressive cells, including regulatory T cells (T-regs), myeloid-derived suppressor cells and tumour-associated macrophages [14,15,16]. In turn, these immunosuppressive cells secrete pro-angiogenic factors that further accelerate uncontrolled tumour angiogenesis and hinder CD8+ cytotoxic T lymphocyte trafficking and infiltration into the TME. Despite well-known effects of VEGF on EC-derived angiogenesis, its modulation of EC immune response is not fully defined.

Bevacizumab, a humanized anti-VEGF monoclonal antibody (mAb), is clinically used for treatment of cancers, including metastatic colorectal, metastatic breast and non-small-cell lung cancers. Clinical findings have shown that Bevacizumab exhibits not only anti-angiogenic efficacy but also contributes to supporting T cell function to enhance antitumor immunity [17,18]. Bevacizumab increased T cell and B cell compartments in patients with metastatic colorectal cancer [19], improved the cytotoxic T lymphocytes response in patients with metastatic non-small-cell lung cancer [20], expanded the extent of CD3+ and CD8+ T cell infiltration in glioblastoma tissues of patients [21] and sustained the circulation of the effector T cells in patients with ovarian cancer [22]. Furthermore, Bevacizumab has been shown to promote dendritic cell (DC) activation [20] and enhance the number of matured DCs in peripheral blood from patients with lung, breast and colorectal carcinoma [17]. In addition to its effects on DCs, Bevacizumab has been shown to restore the TME to an immune supportive state by reducing the proportion of T-regs in the blood of patients with metastatic colorectal cancer [14] or ovarian cancer [22] and decreasing the number of PD-L1 positive tumour cells in glioblastoma patients [21].

Anti-VEGF therapy, therefore, not only reduces excessive angiogenesis and normalises the tumour vessel structure to improve drug perfusion and efficacy, but it also has the potential to reprogram the TME from an immune suppressive to immune permissive microenvironment. These effects are advantageous for immunotherapy and alleviate the resistance to immune checkpoint inhibitors [23,24]. Consequently, combining immune checkpoint blockade with VEGF antagonism is a rational therapeutic strategy for hindering tumour growth. Indeed, following synergistic and positive outcomes (including the increased tumour T cell infiltration and prolonged patient survival), co-administration of immune checkpoint inhibitors and anti-angiogenic therapies has recently made considerable progress in clinical trials, particularly for renal cell and hepatocellular carcinomas and non-small-cell lung cancer. There are over 90 clinical trials presently evaluating the clinical benefit of combining immune checkpoint therapy with anti-angiogenic treatment [25], and to date, Bevacizumab in combination with Atezolizumab against PD-L1 has been FDA approved for treatment of metastatic non-squamous non-small-cell lung cancer [26] and unresectable hepatocellular carcinoma [27].

While increasing clinical evidence has suggested the immunological effects of Bevacizumab on T cells in cancer patients, much less is known about the potential impact of anti-VEGF treatment on vascular ECs, which are potent immune regulator cells that function by forming the blood–tissue barrier in control of the trafficking of various leukocyte populations [28,29,30]. Through their ability to express cytokine receptors, ECs can respond to inflammatory stimuli and elicit multiple activities. These can include surface expression of adhesion molecules such as E-selectin, inter-cellular adhesion molecule-1 (ICAM-1) and vascular cell adhesion molecule-1 (VCAM-1), release of cytokines and chemokines and upregulation of major histocompatibility complex (MHC) proteins, Class I and Class II, which can have a crucial impact on promoting an immune response [31,32,33]. Within the TME, ECs represent a unique structural component critical for tumour angiogenesis and immune tolerance [34,35,36,37].

In the present study, we addressed a research question on what the impact of anti-VEGF treatment on vascular ECs is with a focus on their immune response. To answer this, we investigated the in-vitro modulatory effects of Bevacizumab on the immune response of primary human umbilical cord endothelial cells (HUVECs), a well-established and the most used human primary cellular model generally representing the characteristics of vascular ECs.

## 2. Results

### 2.1. Bevacizumab Increased TNF-α Stimulation of EC Surface Expression of Adhesion Molecules

**Figure 1:**
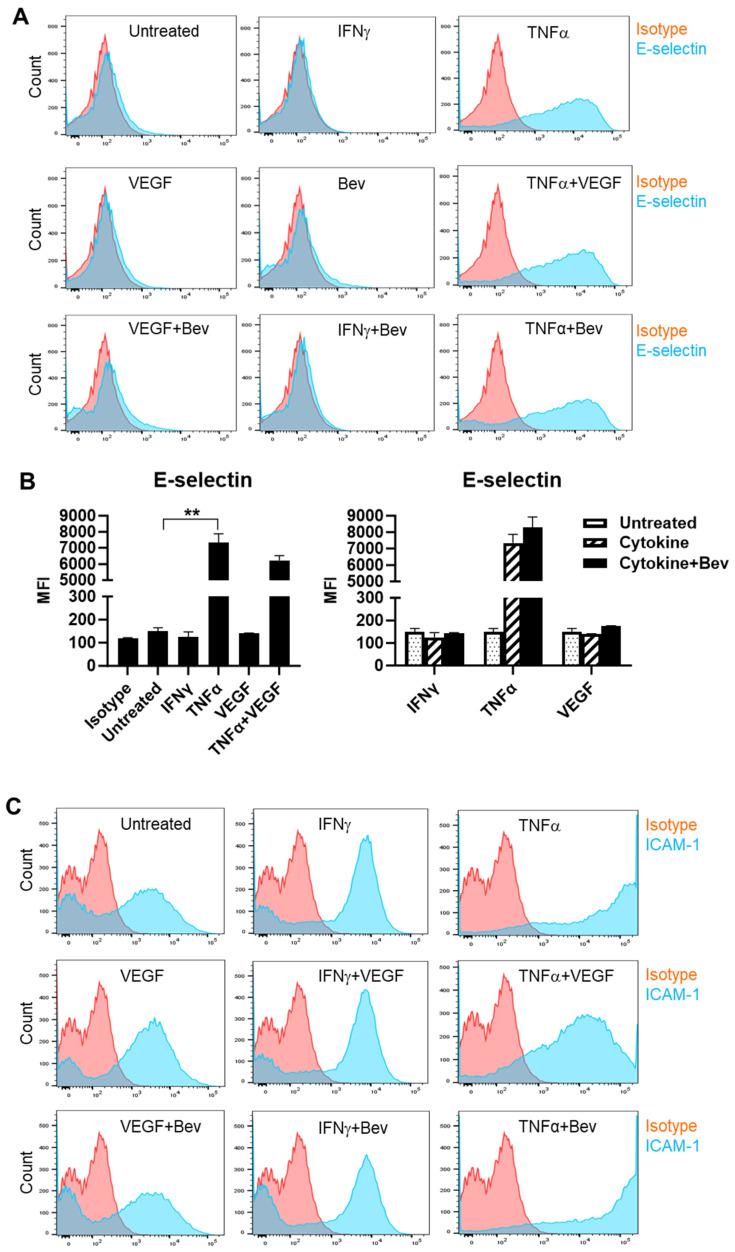

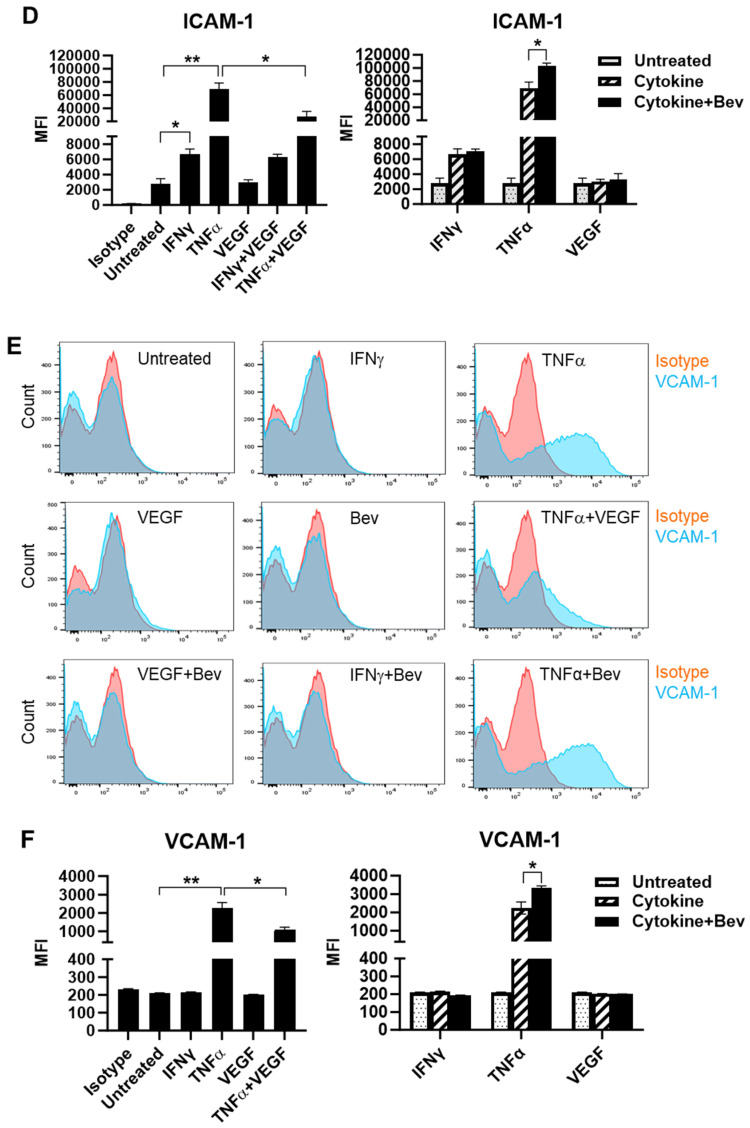
Modulation of Bevacizumab on cytokine effects on surface expression of adhesion molecules. HUVECs were treated without (as untreated control) or with IFN-γ or TNF-α at 10 ng/mL or VEGF at 25 ng/mL in the absence or presence of Bevacizumab at 5 μg/mL for 24 h. The cell surface expression of E-selectin (**A**,**B**), ICAM-1 (**C**,**D**) and VCAM-1 (**E**,**F**) was detected via flow cytometry using specific fluorescence-labelled antibodies alongside isotype-matched antibodies, respectively. Pink histograms represent isotype-matched control staining and blue histograms represent specific antibodies for adhesion molecules staining. MFI indicates the median fluorescence intensity. * *p* < 0.05; ** *p* < 0.01.

To investigate the immune modulatory properties of Bevacizumab on ECs with respect to an early response to inflammatory stimuli or VEGF treatment, HUVECs were treated with IFN-γ, TNF-α or VEGF in the presence or absence of Bevacizumab, and surface expression of adhesion molecules known to be involved in interactions with immune cells was examined at constitutive levels and on stimulation. We found that E-selectin was little expressed on the surface of resting HUVECs compared with isotype controls (Figure 1A,B). However, treatment with TNF-α profoundly induced surface expression of E-selectin, while IFN-γ and VEGF had little effect. It was noticed that VEGF had a tendency to decrease TNF-α induction of E-selectin expression whereas addition of Bevacizumab promoted TNF-α-induced E-selectin expression, although it was non-significant (Figure 1B). In addition, no changes of E-selectin expression were observed on HUVECs after co-treatment with IFN-γ and VEGF. As shown in Figure 1C,D, HUVECs constitutively expressed ICAM-1 at a high level, and both IFN-γ and TNF-α further increased ICAM-1 expression with TNF-α more effective than IFN-γ. VEGF reduced TNF-α stimulation of ICAM-1 expression by 60%, but not IFN-γ. In contrast, Bevacizumab significantly promoted expression of ICAM-1 stimulated by TNF-α (Figure 1D). As with E-selectin, VCAM-1 was hardly detectable on the surface of resting HUVECs (Figure 1E,F). TNF-α, but not IFN-γ, induced surface expression of VCAM-1, and this was decreased by VEGF to 41%. Consistently, Bevacizumab further increased TNF-α induction of VCAM-1 expression by 70% (Figure 1F).

### 2.2. Bevacizumab Promoted Cytokine Upregulation and Reversed VEGF Downregulation of EC Surface Expression of MHC Class I Molecule

**Figure 2:**
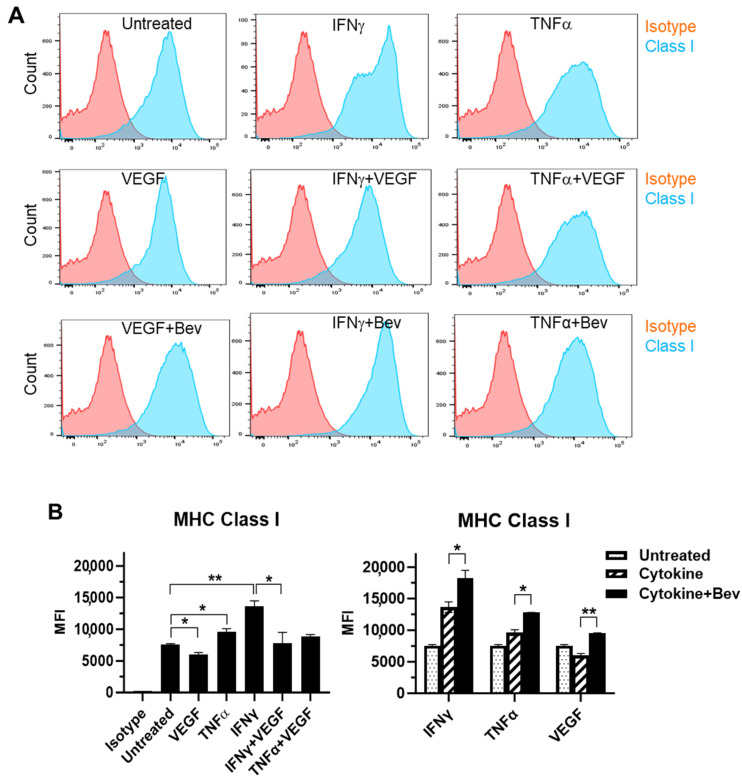

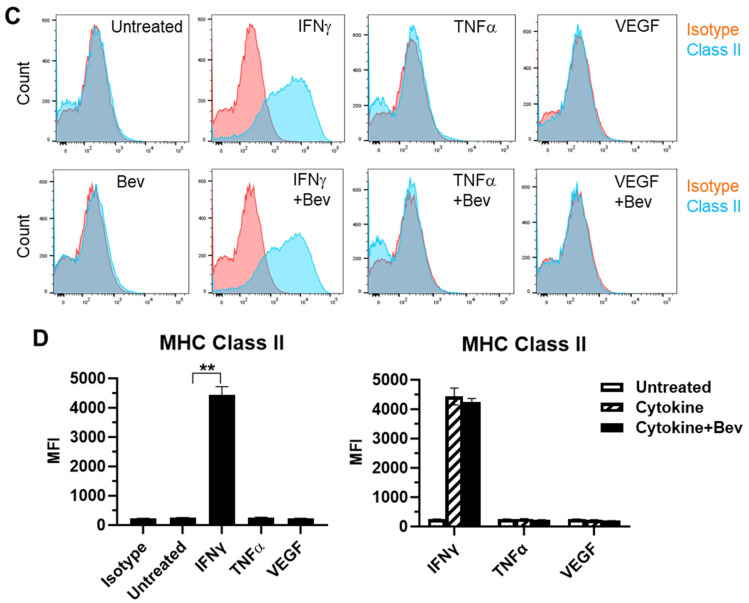
Modulation of Bevacizumab on cytokine effects on surface expression of MHC molecules. HUVECs were treated without (as untreated control) or with IFN-γ or TNF-α at 10 ng/mL or VEGF at 25 ng/mL in the absence or presence of Bevacizumab at 5 μg/mL for 24 h. The cell surface expression of MHC Class I (**A**,**B**) and MHC Class II (**C**,**D**) was detected via flow cytometry using specific fluorescence-labelled antibodies alongside isotype-matched antibodies, respectively. Pink histograms represent isotype-matched control staining and blue histograms represent specific antibodies for MHC molecules staining. MFI indicates the median fluorescence intensity. * *p* < 0.05; ** *p* < 0.01.

We next evaluated the immune modulatory effects of Bevacizumab on EC antigen-presenting cell function (such as the presentation of MHC proteins) in response to inflammatory cytokines. The basal expression of the MHC Class I molecule was evident at a high level on the surface of resting HUVECs (Figure 2A,B). Both IFN-γ and TNF-α increased the surface expression of Class I with IFN being more effective than TNF. This cytokine-stimulated Class I expression was promoted by combining Bevacizumab treatment to a further increase by 34% versus IFN-γ alone and 33% versus TNF-α alone, respectively. On the contrary, VEGF decreased basal and IFN-γ-stimulated levels of Class I expression by 20% and 43%, respectively, but this was blocked by Bevacizumab (Figure 2B). Unlike MHC Class I, MHC Class II was not present on the surface of resting HUVECs (Figure 2C,D). Of the three factors, only IFN-γ induced a pronounced expression of the Class II molecule, and this was not modulated by adding Bevacizumab (Figure 2D).

### 2.3. Bevacizumab Abolished VEGF-Mediated Reduction and Increased TNF-α-Mediated Promotion of PBMC Proliferation

**Figure 3:**
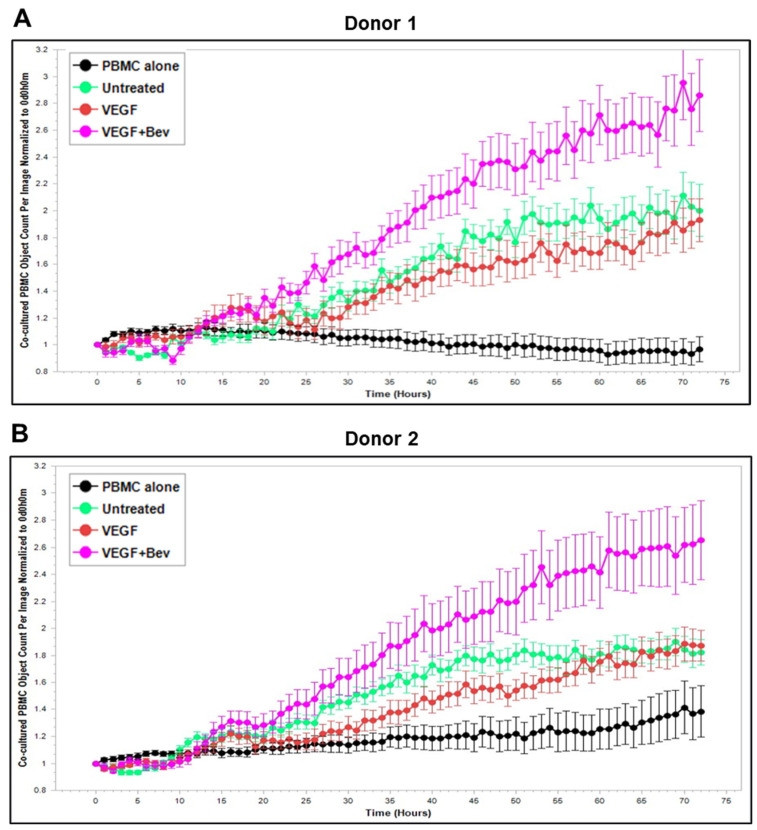

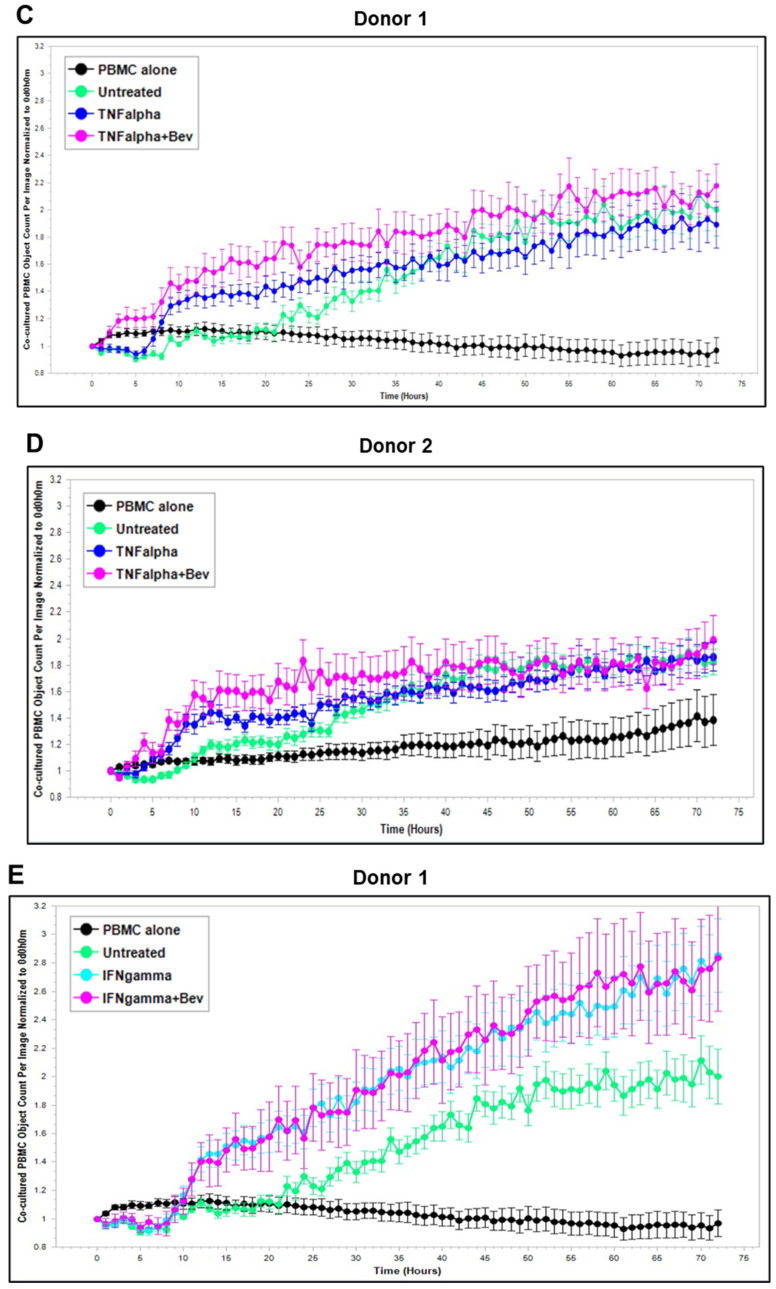

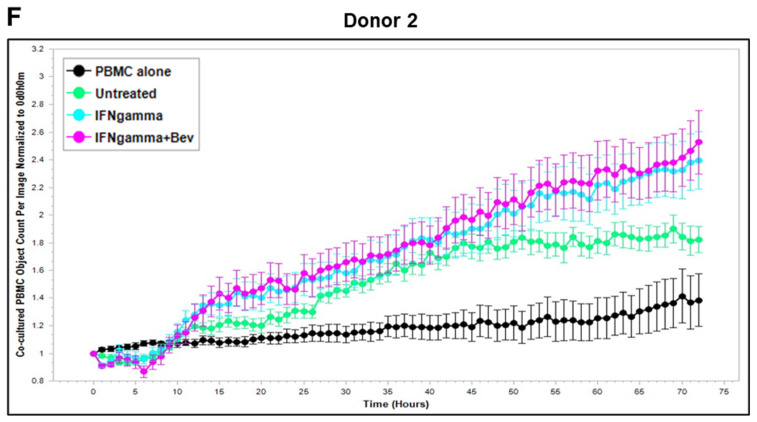
Modulation of Bevacizumab on VEGF inhibition and cytokine activation of proliferation of PBMCs co-cultured with HUVECs in real-time. Proliferation dynamics of PBMCs from donor 1 (**A**,**C**,**E**) and donor 2 (**B**,**D**,**F**) in co-culture on a monolayer of HUVECs treated without (as untreated control) or with 25 ng/mL VEGF (**A**,**B**), 10 ng/mL TNF-α (**C**,**D**), or 10 ng/mL IFN-γ (**E**,**F**) in the absence or presence of 5 μg/mL Bevacizumab over a period of 3 days were monitored using the Incucyte Lice-Cell Analysis System and quantified using the Incucyte^®^ Non-Adherent Cell-by-Cell Analysis Software (https://www.sartorius.com/en/products/live-cell-imaging-analysis/live-cell-analysis-software/incucyte-cell-by-cell-analysis-software, accessed on 5 June 2025) for segmenting phase-contrast images. PBMC alone was used as EC-free untreated control. Representative real-time growth plots of PBMCs are from four independent experiments. Values shown are as the mean ± SD of images from 4 wells per condition. See also Supplementary Videos S1–S7.

Based on these findings, we further explored Bevacizumab modulation at the functional level, i.e., its effects on the interaction and activation of immune cells by ECs under an inflammatory environment. To gain dynamic insights into any functional changes of immune cells during communication with ECs, we developed a new co-culture model for real-time monitoring of the proliferation of activated human peripheral blood monocyte cells (PBMCs) in direct contact with a monolayer of HUVECs using the Incucyte Live-Cell Analysis System [38] and investigated PBMC responses in the absence or presence of VEGF or cytokines and without or with combined Bevacizumab treatment. As shown in Figure 3A,B, exposure of the co-culture of PBMCs from donor 1 or donor 2 with HUVECs to VEGF caused a time-dependent decrease in proliferation of PBMCs on the HUVEC monolayer compared with the untreated control over a 3-day period course, and this was strikingly abolished in the presence of Bevacizumab (Supplementary Videos S1–S3). Contrary to VEGF, TNF-α caused an increase in proliferation of PBMCs on the HUVEC monolayer up to 1.5 days, which was sustained until the end of the time course (Figure 3C,D). Bevacizumab continuously promoted TNF-α stimulation of PBMC proliferation over 3 days (Supplementary Videos S4 and S5). IFN-γ showed a stronger ability than TNF-α to continuously increase the proliferation of PBMCs over the time course, and there was no observed modulation via combination treatment with Bevacizumab (Figure 3E,F; Supplementary Videos S6 and S7). A similar tendency in Bevacizumab modulation of proliferation kinetics of PBMCs in the presence of VEGF or TNF-α was retained in donor 3. Therefore, the findings of PBMC proliferation inhibited by VEGF, stimulated by TNF-α and modulated by Bevacizumab were consistent between individual donors of PBMCs although there was a difference in the magnitude of PBMC growth dynamics (Figure 3A–F), suggesting that Bevacizumab can promote immune cell–EC interactions and increase the sensitivity of co-cultures to the activation by TNF-α for PBMC proliferation.

### 2.4. Effects of Bevacizumab on T Cell Activation

**Figure 4:**
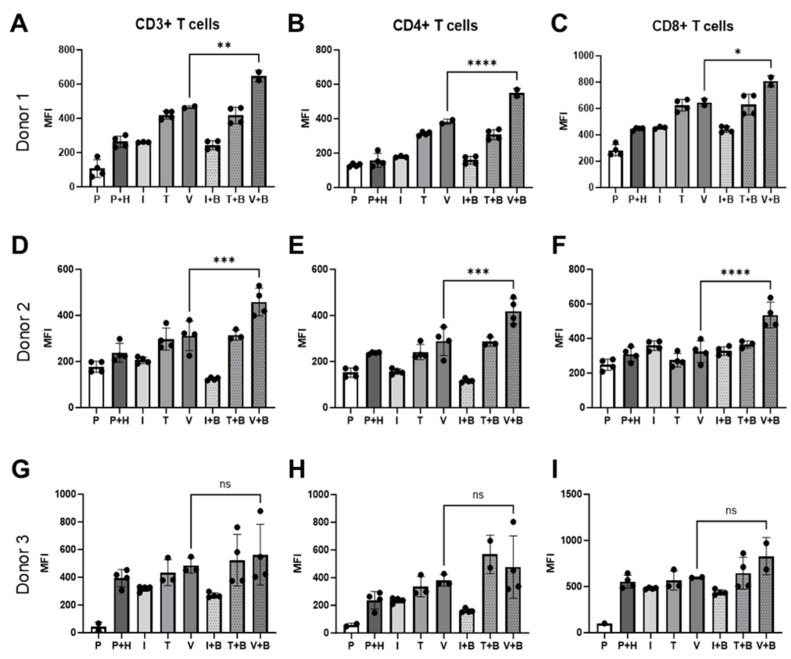
Bevacizumab upregulates activation of VEGF-treated T cells. CD69 expression on CD3+ (**A**,**D**,**G**), CD4+ (**B**,**E**,**H**) and CD8+ (**C**,**F**,**I**) T cells was upregulated by Bevacizumab in HUVEC and PBMC co-cultures exposed to VEGF, but not IFN-γ or TNF-α. PBMCs alone were used as EC-free untreated controls. Values shown are mean ± SEM for three individual donors from at least two separate experimental runs with three replicates. P—PBMCs culture without HUVECs, P+H—untreated HUVECs and PBMCs co-culture, I—IFN-γ-treated HUVECs and PBMCs co-culture, T—TNF-α-treated HUVECs and PBMCs co-culture, V—VEGF-treated HUVECs and PBMCs co-culture, I+B—IFN-γ- and Bevacizumab-treated HUVECs and PBMCs co-culture, T+B—TNF-α- and Bevacizumab-treated HUVECs and PBMCs co-culture, V+B—VEGF- and Bevacizumab-treated HUVECs and PBMCs co-culture. * *p* < 0.05; ** *p* < 0.01, *** *p* < 0.001; **** *p* < 0.0001. ns indicates not statistically significant.

Alongside the Incucyte measurement of PBMC proliferation on the HUVEC monolayer in real-time, we also conducted an end-point flow cytometry analysis of T cells derived from PBMCs co-cultured with HUVECs to investigate Bevacizumab’s modulation on activation of T cells in response to inflammatory cytokines or VEGF. At day 3 of co-culture, Bevacizumab promoted upregulation of cluster of differentiation (CD69), an early activation marker, on pan (CD3+), helper CD4+ and cytotoxic CD8+ T cells (Figure 4) but only when PBMCs were exposed to VEGF. We found that for donor 1 CD69 expression on CD3+ T cells co-cultured with HUVECs (263.5 ± 16.83 MFI) remained unchanged upon IFN-γ stimulation (260.7 ± 1.2 MFI) but increased when TNF-α (419 ± 11.48 MFI) and VEGF (465.5 ± 5.5 MFI) were added to co-cultures. Addition of Bevacizumab to VEGF-treated co-cultures (but not to IFN-γ or TNF-α-treated co-cultures) further increased CD69 expression (646 ± 24 MFI from 465.5 ±5.5 MFI) on CD3+ T cells (Figure 4A). All of this was reflected in an increase of CD69 expression on CD4+ T helper cells for donor 1 from 158 ± 20.41 MFI to 313.5 ± 5.42 MFI and 382.5 ±10.5 MFI when TNF-α and VEGF were added to cultures but not IFN-γ (178.7 ± 2.73 MFI). Further addition of Bevacizumab increased CD69 expression on CD4+ T cells only for co-cultures exposed to VEGF (550.5 ± 18.50) (Figure 4B). Similar results were seen for CD8+ T cell population where the CD69 activation marker was upregulated from 445.3 ± 5.48 MFI to 625 ± 22.16 MFI and 645 ± 21 MFI in co-cultures exposed to TNF-α and VEGF, respectively, but not to IFN-γ (MFI 454.3 ±22.16 MFI). Bevacizumab also upregulated CD69 expression on CD8+ T cells only in co-cultures exposed to VEGF (807.5 ± 29.5 MFI) (Figure 4C).

For donor 2, addition of IFN-γ (205.5 ± 6.76 MFI), TNF-α (297.5 ± 24 MFI) or VEGF (311.3 ± 32.05 MFI) had little effect on CD69 expression on CD3+ T cells co-cultured with HUVECS compared with untreated co-culture (238.3 ± 20.4 MFI). However, we could clearly see that addition of Bevacizumab increased CD69 surface expression on donor 2’s pan (CD3+) T cells in VEGF-stimulated wells (458.3 ±30.04 MFI) (Figure 4D). CD69 expression on CD4+ T helper cells also remained unchanged when TNF-α (241.3 ±16.38 MFI) and VEGF (288.3 ±30.86 MFI) were added to the wells, but it slightly decreased when IFN-γ (156.5 ± 5.95 MFI) was added in comparison to untreated co-culture (236.8 ± 2.36 MFI). Bevacizumab treatment increased T cell activation marker expression in co-cultures exposed to VEGF (418.8 ± 27.57 MFI) but not to TNF-α (286.3 ± 11.39 MFI) or IFN-γ (117 ± 4.26 MFI) (Figure 4E). Addition of IFN-γ (358.8.5 ± 13.98 MFI), TNF-α (274.5 ± 19.45 MFI) or VEGF (324.3 ± 31.35 MFI) had no effect on CD69 expression on CD8+ T cells co-cultured with HUVECs compared with untreated co-culture (309.3 ± 20.85 MFI), but as in the case of other T cell subpopulations and in donor 1, treatment with Bevacizumab increased CD69 surface expression on donor 2’s cytotoxic T cells in VEGF-stimulated wells (535.5 ± 37.4 MFI) (Figure 4F). The same trend of T cell activation could be seen for donor 3 (Figure 4G–I), but in this case, it likely reflects a more mispatched combination of PBMCs and HUVECs, as co-culture of the two resulted in significantly increased expression of CD69 on CD3+ T cells (from 42.85 ± 24.45 MFI to 393 ± 31.59 MFI), on CD4+ T cells (from 57.20 ± 9.4 MFI to 235.5 ± 31.61 MFI) and on CD8+ T cells (from 100 ± 0 MFI to 552.5 ± 35.45 MFI) in comparison to other co-cultures (Figure 4A–F). Similar to previous donors, the addition of cytokines had little effect on CD69 expression on CD3+ T cells (IFN-γ 318.3 ± 9.7 MFI; TNF-α 434 ± 54.01 MFI; VEGF 485.3 ± 31.87 MFI), CD4+ T cells (IFN-γ 235.3 ± 9.3 MFI; TNF-α 335.3 ± 42.43 MFI; VEGF 380 ± 24 MFI) or CD8+ T cells (IFN-γ 479.8 ± 4.5 MFI; TNF-α 566 ± 59.52 MFI; VEGF 599.5 ± 3.5 MFI) co-cultured with HUVECs compared with untreated co-culture. The addition of Bevacizumab showed a similar trend in increasing CD69 expression on CD3+ T cells (563 ± 109.8 MFI), CD4+ T cells (476.8 ± 112.7 MFI) and CD8+T cells (828.5 ± 142.5 MFI), but it was not statistically significant for this donor due to higher intra experimental variability for this treatment group (Figure 4G–I).

There were no differences in expression of later T lymphocyte receptor (TCR)-activation marker CD25+ for pan, cytotoxic or helper T cells between any of the co-culture conditions at day 3. CD25 is an IL-2RA, so it is likely that the activation of T cells measured using this marker would be only evident in co-cultures set up for longer than 3 days.

### 2.5. Effects of Bevacizumab on T Cell Proliferation and Integrin Expression

**Figure 5:**
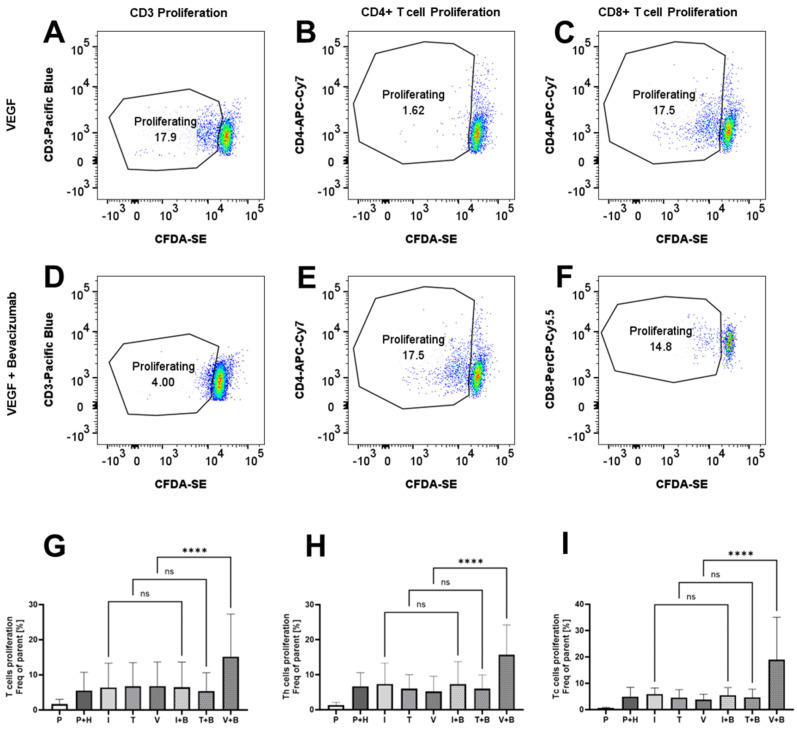
Bevacizumab promotes VEGF-treated T cell proliferation. CD3+ (**A**,**D**,**G**), CD4+ (**B**,**E**,**H**) and CD8+ (**C**,**F**,**I**) T cells in HUVEC and PBMC co-cultures exposed to VEGF, but not IFN-γ or TNF-α, showed increased proliferation following Bevacizumab treatment. PBMCs alone were used as EC-free untreated controls. Representative flow cytometry proliferation plots for different T cell populations for VEGF-treated co-cultures (**A**–**C**) and VEGF + Bevacizumab co-cultures (**D**–**F**). Values shown in graphs (**G**–**I**) are mean ± SEM for three individual donors from three separate experimental runs with three technical replicates each. P—PBMCs culture without HUVECs, P+H—untreated HUVECs and PBMCs co-culture, I—IFN-γ-treated HUVECs and PBMCs co-culture, T—TNF-α-treated HUVECs and PBMCs co-culture, V—VEGF-treated HUVECs and PBMCs co-culture, I+B—IFN-γ- and Bevacizumab-treated HUVECs and PBMCs co-culture, T+B—TNF-α- and Bevacizumab-treated HUVECs and PBMCs co-culture, V+B—VEGF- and Bevacizumab-treated HUVECs and PBMCs co-culture. **** *p* < 0.0001. ns indicates not statistically significant.

We could see that Bevacizumab had a promoting effect on the proliferation of PBMCs from Incucyte data, but we also wanted to check whether this is reflected in the promotion of T cell proliferation. We stained PBMCs with CFDA-SE and, using flow cytometry dye dilution assay, we measured CD3+, CD4+/CD3+ and CD8+/CD3+ T cell proliferation in 3-day HUVEC/PBMC co-cultures. Similar to the immunological bioassays, the data here in Figure 5 were variable, reflecting some donor variability in the levels of T cell proliferation. CD3+, CD4+ or CD8+ T cell proliferation was unaffected by the addition of IFN-γ (6.39 ± 2.82% for CD3+ T cells, 7.36 ± 2.42% for CD4+ T cells and 5.85 ± 0.96% for CD8+ T cells), TNF-α (6.74 ± 2.74% for CD3+ T cells, 5.98 ± 1.64% for CD4+ T cells and 4.52 ± 1.23% for CD8+ T cells) or VEGF (6.79 ± 2.81% for CD3+ T cells, 5.19 ± 1.72% for CD4+ T cells and 3.78 ± 0.83% for CD8+ T cells) compared to untreated co-cultures (5.49 ± 2.13% for CD3+ T cells, 6.63 ± 1.6% for CD4+ T cells and 4.96 ± 1.44% for CD8+ T cells), but we noted that Bevacizumab promotes VEGF-treated T cell proliferation of pan (CD3+), CD4+ and CD8+ T cells (Figure 5). The mean frequency of proliferating cells increased to 15.18 ± 4.95% for CD3+ T cells, 15.68 ± 3.5% for CD4+ T cells and 18.98 ± 6.57% for CD8+ T cells when Bevacizumab was added to VEGF-exposed HUVEC/PBMC co-cultures. No effect was seen when Bevacizumab was added to co-cultures stimulated with IFN-γ or TNF-α at day 3 of the experiments.

**Figure 6:**
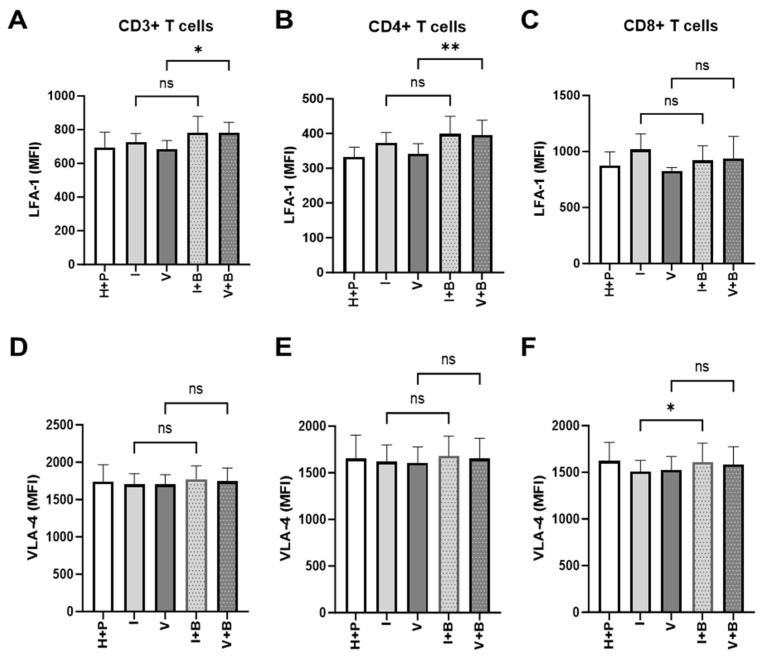
Bevacizumab upregulates expression of lymphocyte function-associated antigen 1 (LFA-1) on VEGF-treated CD4+ T helper cells. CD3+ (**A**) and CD4+ (**B**) T cells but not CD8+ T cells (**C**) in HUVEC and PBMC co-cultures exposed to VEGF showed increased surface expression of LFA-1 integrin involved in T cell migration. IFN-γ-primed co-cultures remained unaffected by Bevacizumab treatment. The levels of very late activation antigen-4 (VLA-4) integrin were unaffected when Bevacizumab was added to co-cultures (**D**–**F**). Values shown are mean ± SEM for three individual donors from three separate experimental runs with three technical replicates each. P+H—untreated HUVECs and PBMCs co-culture, I—IFN-γ-treated HUVECs and PBMCs co-culture, V—VEGF-treated HUVECs and PBMCs co-culture, I+B—IFN-γ- and Bevacizumab-treated HUVECs and PBMCs co-culture, V+B—VEGF- and Bevacizumab-treated HUVECs and PBMCs co-culture. * *p* < 0.05; ** *p* < 0.01. ns indicates not statistically significant.

The inability of T cells to reach tumour cells is an important aspect of tumour resistance to cancer immunotherapy. Having observed Bevacizumab promotion of TNF-α induction of expression of ICAM-1 and VCAM-1 on the surface of HUVECs above, next we looked at the signs of effective priming of T cells to cross the blood vessel wall into the tumour microenvironment. To do that, we measured the effect of Bevacizumab on the regulation of T cell integrins, lymphocyte function-associated antigen-1 (LFA-1) and very late activation antigen-4 (VLA-4), that facilitate endothelial adhesion to ICAM-1 and VCAM-1. Bevacizumab showed the ability to upregulate expression of LFA-1 but not VLA-4 on pan and CD4+ T cells but not CD8+ T cells in a 3-day HUVEC/PBMC co-culture (Figure 6). The addition of Bevacizumab to co-cultures resulted in an increase of LFA-1 MFI from 685.8 ±28.58 (for VEGF treatment alone) to 782.2 ± 35.39 MFI on T cells and from 341.2 ± 17.28 to 395.1 ± 25.08 on T helper cells (CD4+ T cells). The expression of LFA-1 on CD8+ cytotoxic T cells increased from 827.8 ± 16.42 to 935.9 ± 114.7, but it did not reach a significant difference due to donor-to-donor variability. Additions of IFN-γ (725 ± 29.99 MFI for CD3+ T cells, 373.2 ± 17.27 MFI for CD4+ T cells and 1018 ± 80.76 MFI for CD8+ T cells) or VEGF (685.8 ± 28.58 MFI for CD3+ T cells, 341.2 ± 17.28 MFI for CD4+ T cells and 827.8 ± 16.42 MFI for CD8+ T cells) to co-cultures or further treatment with Bevacizumab to co-cultures exposed to IFN-γ (783.7 ± 55.43 MFI for CD3+ T cells, 399.4 ± 29.04 MFI for CD4+ T cells and 920.8 ± 74.99 MFI for CD8+ T cells) showed no effect on LFA-1 expression on T cells or any of the T cell subsets (Figure 6A–C) when compared to untreated co-cultures (695.3 ± 51.97 MFI for CD3+ T cells, 332.1 ± 20.13 MFI for CD4+ T cells and 872.6 ± 71.51 MFI for CD8+ T cells). We also looked at expression of VLA-4 on T cells in co-cultures subjected to VEGF and IFN-γ with or without Bevacizumab. VLA-4 expression on CD3+ or CD4+ T cells was not promoted by Bevacizumab treatment when VEGF was added to co-cultures (Figure 6D,E) and there was only a slight increase in VLA-4 expression on cytotoxic T cells from 1509 ± 68.75 MFI to 1611 ± 115.5 MFI when Bevacizumab was added to IFN-γ-exposed co-cultures (Figure 6F).

## 3. Discussion

The present study showed that Bevacizumab enhanced TNF-α stimulation of endothelial surface expression of adhesion molecules ICAM-1 and VCAM-1, while VEGF reduced TNF-α stimulation of surface expression of both molecules on HUVECs. Bevacizumab promoted IFN-γ and TNF-α upregulation of surface expression of the MHC Class I molecule and reversed VEGF downregulation of Class I on HUVECs. Functionally, Bevacizumab continuously abolished VEGF attenuation and increased TNF-α stimulation of proliferation of PBMCs in co-culture on the monolayer of HUVECs during 3-day real-time monitoring of growth dynamics of PBMCs. Consistently, Bevacizumab elevated proliferation of total CD3+, CD8+ cytotoxic and CD4+ helper T cells in the presence of VEGF after 3-day co-cultures with HUVECs. Furthermore, the surface expression of the activation marker CD69 on these T cells was raised via Bevacizumab treatment. Additionally, Bevacizumab showed the ability to upregulate expression of LFA-1 on CD3+ and CD4+ T cells. Taken together, these data indicate that Bevacizumab treatment can promote the immune response of human vascular ECs and immune cell–EC interactions to sensitise the proliferation of PBMCs and T cells. Importantly, since previous clinical studies have shown the immune effects of Bevacizumab on T cells, but little information on the impact of Bevacizumab on immunological reactions of vascular ECs, this investigation, for the first time, through the angle of ECs and their crosstalk with immune cells, provides the molecular insights into the property of Bevacizumab-promoted endothelial immunity and fills in the mechanistic gap within the immune-supportive portfolios of anti-VEGF therapy.

The diverse functions of VEGF in tumour angiogenesis and immune cell suppression are well described [7,8], but its exact role in direct regulation of EC response to inflammatory stimuli at the phenotypic and functional levels is still largely unknown. Our data show distinct effects between VEGF and inflammatory cytokines on HUVEC surface expression of adhesion molecules. VEGF neither induced surface expression of E-selectin and VCAM-1, nor affected the basal level of ICAM-1 expression. However, it inhibited TNF-α induction of surface expression of ICAM-1 and VCAM-1. This supports previous reports of VEGF reduction of TNF-α-induced upregulation of ICAM-1 and VCAM-1 in cultured ECs and tumour-associated ECs isolated from melanoma tissues [39,40]. Consistent with its immunological effects on T cells [17], we observed that Bevacizumab enhanced TNF-α induction of expression of both ICAM-1 and VCAM-1. This agrees with the previous study by Wu et al. [40], which showed that Bevacizumab blocked VEGF inhibition of TNF-α-induced expression of ICAM-1 and VCAM-1 in the melanoma-associated ECs. Furthermore, Bevacizumab upregulation of surface expression of LFA-1 on T cells was revealed. As LFA-1 is a receptor for ICAM-1, it is crucial for firm adhesion of immune cells to ECs through its direct molecular interaction with endothelial ICAM-1 for stable interaction and migration, facilitating immune cell trafficking [41]. Therefore, our finding suggests that anti-VEGF treatment can modulate expression of adhesion molecules in favour of the EC immune response required for recruitment of immune cells.

Vascular ECs can also exhibit a semi-professional antigen-presenting cell function and have been shown to selectively recruit antigen-specific CD8+ cytotoxic and CD4+ helper T cells through their ability to express MHC Class I and Class II molecules [42]. We revealed that in contrast to IFN-γ and TNF-α augmentation, VEGF decreased surface expression of MHC Class I, whereas Bevacizumab overturned VEGF downregulation and enhanced cytokine upregulation of this molecule. These results indicate that targeting of VEGF can modulate surface expression of MHC Class I to promote the EC-driven immune response required for communication and recruitment of CD8+ T cells. Given that the productive CD8+ T cell-mediated anti-tumour immunity is dependent on the ability to traffic to the TME [5], this study supports the clinical findings, which have demonstrated the augmented expression of MHC Class I and the improvement of CD8+ T cell response and infiltration in tumour tissues of patients following VEGF blockade via Bevacizumab [20,21,43].

Immune cells recruitment by activated ECs involves a close interaction between immune cells and ECs, which can induce functional changes in immune cells such as activation and proliferation to prepare them for crossing the vessel wall into the local tissue [44]. To capture these dynamic events for better understanding, we monitored real-time proliferation of PBMCs on a monolayer of HUVECs. We found that VEGF continuously slowed down the proliferation of PBMCs co-cultured with HUVECs, and this was counteracted by Bevacizumab over the 3-day co-culture period, suggesting Bevacizumab neutralisation of VEGF inhibition of endothelial–immune cell interactions. This is consistent with VEGF downregulation of cytokine-induced expression of adhesion molecules ICAM-1 and VCAM-1 as well as MHC Class I, which are required for endothelial–immune cell interactions, and with VEGF inhibition of the responsiveness to proinflammatory cytokines of immune cells. Bevacizumab continuously promoted TNF-α stimulation of PBMC proliferation, and this is in line with its promotion of TNF-α-induced expressing of adhesion molecules. Our findings also support previous clinical reports, which showed VEGF suppression of activation and recruitment of T cells [8], while Bevacizumab promoted growth and infiltration of T cells into tumour tissues of cancer patients [17,18].

Using flow cytometric analysis of T cells collected from the 3-day co-culture of PBMCs on the HUVEC monolayer, we found TNF-α upregulation of surface expression of CD69 as an indicator of early T cell activation, which was consistent with previous reports [45,46,47]. Bevacizumab treatment promoted the surface expression of CD69 and proliferation of CD8+ and CD4+ T cells. The finding is supportive of the dynamics of Bevacizumab effects on PBMC–EC interaction and activation in real-time and consistent with its upregulation of the surface expression of the MHC Class I molecule on ECs as revealed by the present study. Our data confirms previous clinical reports of the increased number of CD8+ and CD4+ T cells in metastatic colorectal cancer patients following Bevacizumab treatment [19]. It is conceivable that the dual role of Bevacizumab on ECs and T cells can facilitate the infiltration of CD8+ T cells into tumours and enable direct tumour cell killing as they are the main lymphocytes in cell-mediated antitumor immunity [48,49].

Immunological cell-based bioassays are essential for advancing scientific and biomedical investigations, but they can be highly variable because of multiple biological, technical and microenvironmental factors [50], especially in co-cultures of primary PBMCs with vascular ECs. It is important to acknowledge that our finding of no changes in the proliferative responsiveness of T cells following treatment with TNF-α or VEGF does not agree with previous reports of TNF-α stimulation [51] and VEGF inhibition of T cell proliferation [11]. This discrepancy may be due to a range of factors. These may include the differences in the bioassay formats (T cells alone or in co-cultures), the variability between PBMC donors and variable alloantigen-reactivity due to MHC mismatch of co-cultures as well as the cytokine network complexity resulting from intricate cytokine interactions between endogenous and exogenous (added treatment) molecules. It is also worth noting that there were no correlations between our Incucyte and flow cytometric data in the proliferation of PBMCs and T cells. This is likely a reflection of other contributing factors such as the bioassay detection sensitivity, the timing of sample collection and the readouts from dynamic ranges or at the end point. For example, in the Incucyte real-time monitoring experiments, TNF-α and VEGF displayed opposing effects on PBMC proliferation, particularly during days 1 and 2 of co-cultures, while they showed little effects via flow cytometric analysis of T cells collected from co-cultures at the end of day 3. Additionally, the potential proliferation of non-T cells within PBMCs may also contribute to varied readouts between Incucyte and flow cytometry where CD3+ T cells only are gated for the data analysis.

## 4. Materials and Methods

### 4.1. Cell Culture

Primary HUVECs (Cellworks, Buckingham, UK, product code: ZHC-2102) were obtained from Caltag Medsystems (Buckingham, UK). The lot-specific certificate of analysis was provided. HUVECs were cultured in endothelial cell growth medium (EGM; Lonza Biologics Plc, Cambridge, UK, catalog number CC3124) supplemented with bovine brain extracts, 2% fetal bovine serum and gentamicin/amphotericin-B (Lonza Biologics Plc, Cambridge, UK) in an incubator at 37 °C with 5% CO_2_ and passaged via trypsinization (trypsin/EDTA solution, Lonza Biologics Plc). HUVECs at passages from 3 to 8 were used for experiments.

### 4.2. Isolation of Human Peripheral Blood Mononuclear Cells

Peripheral blood mononuclear cells (PBMCs) were isolated from three different donor-leucocyte cones obtained from the National Health Service Blood and Transplant unit (NHSBT), using Lymphoprep Histopaque (07851, STEMCELL Technologies, Cambridge, UK) density gradient centrifugation according to manufacturer’s instructions. Briefly, the cone content was diluted with PBS up to 50 mL volume and overlaid on top of Lymphoprep at 2:1 ratio and centrifuged at 800× *g* for 20 min with slow acceleration and no brakes. Upon density gradient separation, the top plasma layer was removed and the opaque interphase containing PBMCs was gently collected and washed in PBS. Contaminating red blood cells were lysed using ACK lysis buffer (A1049201, Gibco: Thermo Fisher Scientific, Waltham, MA, USA) at room temperature for 5 min. PBMCs at a density of 10^7^–10^8^ cells/mL were frozen in 10% DMSO/FCS at −80 °C with subsequent long-term storage in liquid nitrogen.

### 4.3. EC Surface Immunostaining for Flow Cytometric Analysis

ECs with or without cytokine treatment were harvested using trypsin-EDTA solution and collected via centrifugation. Following initial treatment with the human Fc receptor blocking reagent (Bio-Techne, Abingdon, UK, 1-001-A), EC surface expression of E-selectin, ICAM-1 and VCAM-1 was detected via staining with PE-conjugated anti-E-selectin IgG1 mAb (Bio-Techne, FAB6169P), APC-conjugated anti-ICAM-1 IgG_1_ kappa mAb (eBioscience™, 17-0549-42, supplied by Thermo Fisher Scientific, Hemel Hempstead, UK) and PE-conjugated anti-VCAM-1 IgG2A mAb (Bio-Techne, FAB5649P), respectively. In parallel, the cells were stained with the corresponding isotype-matched control mAbs (Bio-Techne, IC002P or IC003P; eBioscience™, 17-4714-41), respectively. For cell surface expression of MHC Class I and Class II molecules, PE-conjugated anti-human MHC Class I/HLA IgG_2A_ mAb (Bio-Techne, FAB7098P) and APC-conjugated anti-human MHC Class II/HLA-DR IgG_1_ mAb (Bio-Techne, FAB4869A) were used for staining respectively along with the respective isotype-matched control mAb (Bio-Techne, IC003P or IC002A). The stained cells were examined via flow cytometry using FACSCanto II (BD Biosciences, San Jose, CA, USA) by counting 30,000 events. Data were analysed using FACSDiva (version 6) and FlowJo (version 10) software and expressed as histogram overlayers and quantitative bar graphs with median fluorescence intensity (MFI), respectively.

### 4.4. Real-Time Monitoring of Proliferation of PBMCs Co-Cultured on a Monolayer of ECs

Human PBMCs were plated at a seeding density of 10,000 cells per well on a monolayer of HUVECs in a 96-well plate and the co-cultures were incubated without or with VEGF at 25 ng/mL, TNF-α at 10 ng/mL or IFN-γ at 10 ng/mL (Bio-Techne, catalog number 293-VE, 210-TA, 285-IF respectively) in the absence or presence of Bevacizumab at 5 µg/mL in EGM. PBMCs were also seeded at the density of 10,000 cells per well on 0.01% poly-L-ornithine solution (Sigma-Aldrich, Dorset, UK, Catalog number P4957) coated wells in the absence of ECs to serve as EC-free untreated control. The cell-containing plate was placed into an Incucyte chamber at 37 °C with 5% CO_2_ for real-time visualization and automated measurement of PBMC proliferation using the Incucyte Live-Cell Analysis System with the phase contrast channel and non-adherent cell-by-cell scan every 1 h over a 3-day period.

### 4.5. PBMC-HUVEC Co-Cultures for Flow Cytometry Analysis of T Cell Activation and Proliferation

Frozen PBMCs from three individual donors were thawed in a water bath, washed in RPMI medium and suspended at 10^6^ cells/mL in RPMI medium substituted with 10%FCS, L-Glutamine and antibiotics. PBMCs were rested for 48 h before Vybrant CFDA-SE (V12883, Invitrogen: Thermo Fisher Scientific, Waltham, MA, USA) labelling according to the manufacturer’s instructions. Primary HUVECs were cultured as described above to establish a confluent monolayer in 96-well plates. CFDA-SE-labelled PBMCs were resuspended in EGM media at 10^6^/mL and added to a HUVEC monolayer treated with VEGF, TNF-α and IFN-γ, with or without addition of Bevacizumab as described above for 3 days. Dye dilution was used to establish T cell proliferation for pan, CD4+ and CD8+ T cells. PBMCs lifted from HUVEC co-cultures were stained with the following anti-human fluorescently labelled mAb: anti-CD3 Pacific Blue (BD Biosciences, San Jose, CA, USA), Cat No. 558117), anti-CD4 APC-Cy7 (BD Biosciences, San Jose, CA, USA, Cat No. 557871), anti-CD8 PerCP Cy5,5 (BD Biosciences, San Jose, CA, USA, Cat No. 560662) or the corresponding fluorescently labelled isotope-matched controls (for anti-CD3: Mouse BALB/c IgG1, κ, BD Biosciences, San Jose, CA, USA, Cat No. 558120; for anti-CD4: Mouse IgG1, κ BD Biosciences, San Jose, CA, USA, Cat No. 557873; for anti-CD8: Mouse IgG1, κ, BD Biosciences, San Jose, CA, USA, Cat No. 550795), according to the manufacturer’s recommendations. PBMCs were also stained for expression of activation markers on T cells. Cells were stained with the following anti-human fluorescently labelled mAb: anti-CD69 PE-Cy7 (BD Biosciences, San Jose, CA, USA, Cat No. 560712), anti-CD25 PE (BD Biosciences, San Jose, CA, USA, Cat No. 567214), anti-LFA-1-APC (BioLegend, San Diego, CA, USA, Cat. No. 363410) and anti-CD49d-PE (BD Biosciences, San Jose, CA, USA, Cat No.555503) or the corresponding fluorescently labelled isotope-matched controls (for anti-CD69: Mouse IgG1, κ BD Biosciences, San Jose, CA, USA, Cat No. 557872; for anti-CD25: Mouse BALB/c IgG1, κ clone M-A251 BD Biosciences, San Jose, CA, USA, Cat No. 554680; for anti-LFA-1: Mouse IgG1, κ BioLegend, San Diego, CA, USA, Cat No. 400121; for anti-CD49d: Mouse IgG1, κ, BD Biosciences, San Jose, CA, USA, Cat No. 555749), according to manufacturer’s recommendations. Live/Dead far red or aqua stain (Invitrogen: Thermo Fisher Scientific, Waltham, MA, USA, Cat. No. L10120 or L34957) was used for dead cell discrimination. FMO controls were used to gate live singlets. Supplementary Figure S1 shows the gating strategy used for assessment of T cell activation and Supplementary Figure S2 for the assessment of T cell proliferation. Stained cells were examined via flow cytometry using FACSCanto II or BDFortessa (BD Biosciences, San Jose, CA, USA) by counting 10,000 live T cells. Data were analysed using FACSDiva (version 6) and FlowJo (version 10) software and expressed as histogram overlayers and quantitative bar graphs with median fluorescence intensity (MFI) or cell frequencies (%). Presented data are from three experimental runs on three different PBMC donors using three technical repeats in each run.

### 4.6. Statistical Analysis

Data were analysed using GraphPad Prism (version 10) statistical packages. Differences between two groups were assessed using the unpaired *t* test or unpaired *t* test with Welch’s correction where appropriate. Differences among three or four groups were assessed using one-way analysis of variance (ANOVA) with Bonferroni’s multiple comparison tests. Bar graphs represent means ± SEM determined from the results of three independent experiments each performed in three or four replicates unless where stated. A value of *p* < 0.05 was taken as statistically significant.

## 5. Conclusions

The present study advances towards a profound understanding of the molecular mechanisms underlying anti-VEGF therapy-induced immunity of vascular ECs through providing immune-supportive conditions for ECs and their crosstalk with immune cells to facilitate the recruitment of immune cells across the vascular barrier. Thus, it extends beyond the inhibition of angiogenesis by anti-VEGF mAbs. Furthermore, this investigation sheds some light on the strong rationale regarding the beneficial combination of VEGF-targeted therapeutics with immunotherapy to enhance the clinical efficacy and mitigate the drug resistance seen with single-agent use in the combat against cancers.

## Data Availability

The original contributions presented in this study are included in the article. Further inquiries can be directed to the corresponding author.

## Supplementary Materials

The following supporting information can be downloaded at: https://www.mdpi.com/article/10.3390/ijms26136280/s1.

## Abbreviations

The following abbreviations are used in this manuscript:

CTLA-4	Cytotoxic T lymphocyte antigen 4
PD-1	Programmed cell death protein 1
PD-L1	Programmed cell death ligand 1
VEGF	Vascular endothelial growth factor
mAb	Monoclonal antibody
ICAM-1	Inter-cellular adhesion molecule-1
VCAM-1	Vascular cell adhesion molecule-1
MHC	Major histocompatibility complex
HUVEC	Human umbilical cord endothelial cell
PBMC	Peripheral blood mononuclear cell
LFA-1	Lymphocyte function-associated antigen-1
VLA-4	Very late activation antigen-4
