# Supplementary material for: Promoting Immune Response of Human Vascular Endothelial Cells by Bevacizumab: Insights into the Immune Supportive Role of Anti-VEGF Therapy
Source: Int J Mol Sci. 2025 Jun 29;26(13):6280. doi: 10.3390/ijms26136280 (PMC12250103; doi:10.3390/ijms26136280)
Supplement: Supplementary file 1 [file ijms-26-06280-s001.zip › Supplementary Figures_Jia et al.pptx]

## Slide 1
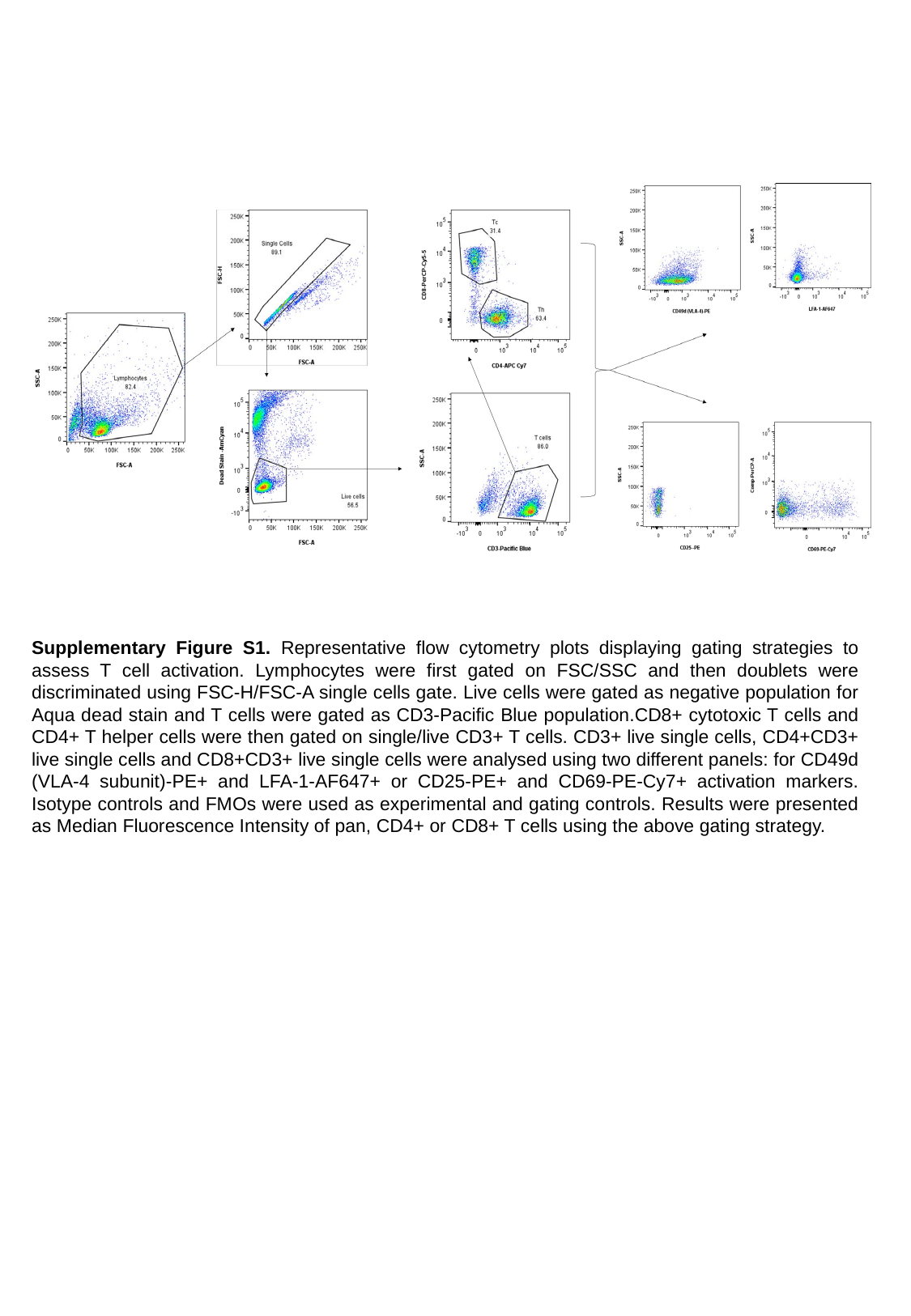

Supplementary Figure S1. Representative flow cytometry plots displaying gating strategies to assess T cell activation. Lymphocytes were first gated on FSC/SSC and then doublets were discriminated using FSC-H/FSC-A single cells gate. Live cells were gated as negative population for Aqua dead stain and T cells were gated as CD3-Pacific Blue population.CD8+ cytotoxic T cells and CD4+ T helper cells were then gated on single/live CD3+ T cells. CD3+ live single cells, CD4+CD3+ live single cells and CD8+CD3+ live single cells were analysed using two different panels: for CD49d (VLA-4 subunit)-PE+ and LFA-1-AF647+ or CD25-PE+ and CD69-PE-Cy7+ activation markers. Isotype controls and FMOs were used as experimental and gating controls. Results were presented as Median Fluorescence Intensity of pan, CD4+ or CD8+ T cells using the above gating strategy.

## Slide 2
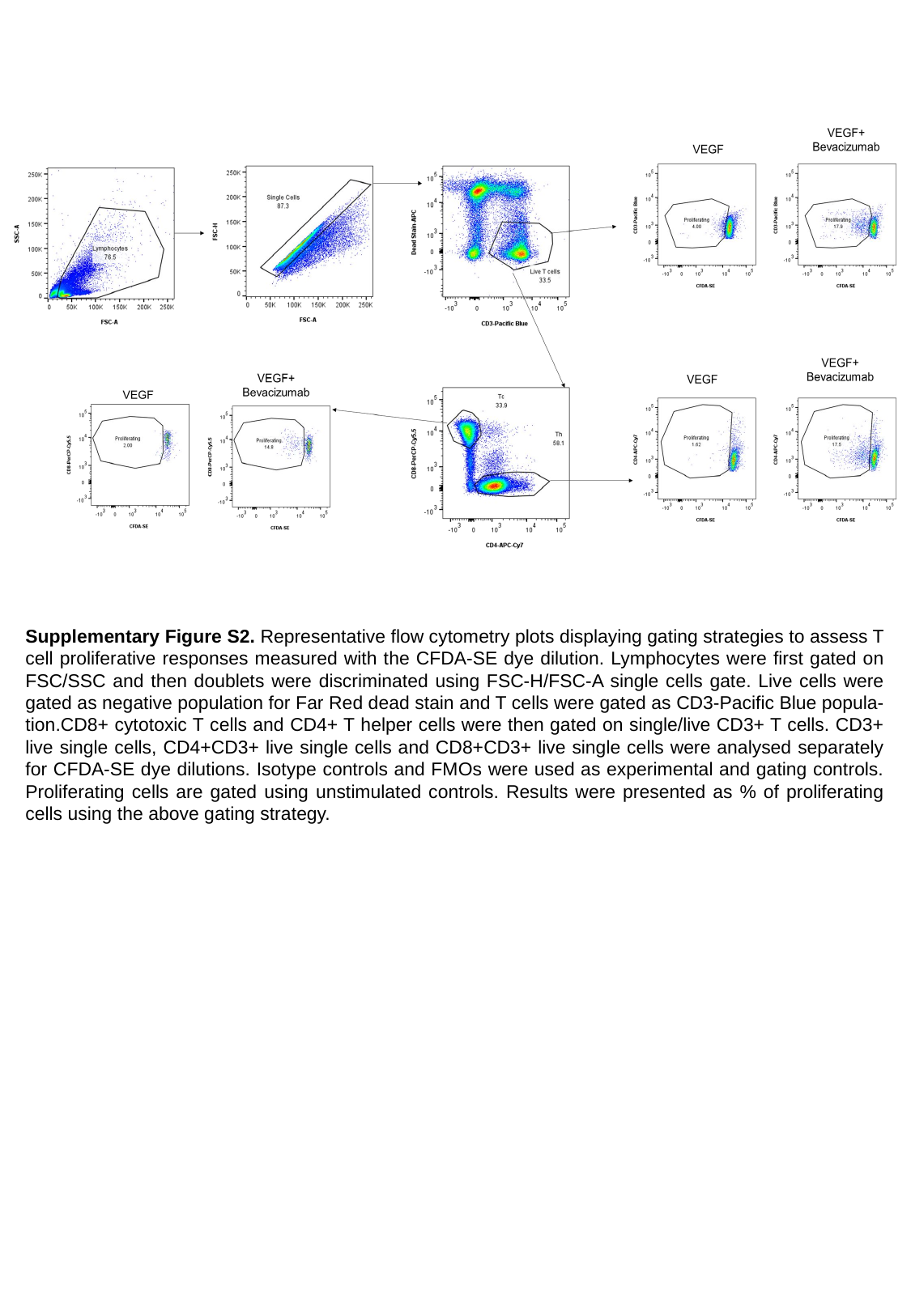

Supplementary Figure S2. Representative flow cytometry plots displaying gating strategies to assess T cell proliferative responses measured with the CFDA-SE dye dilution. Lymphocytes were first gated on FSC/SSC and then doublets were discriminated using FSC-H/FSC-A single cells gate. Live cells were gated as negative population for Far Red dead stain and T cells were gated as CD3-Pacific Blue popula-tion.CD8+ cytotoxic T cells and CD4+ T helper cells were then gated on single/live CD3+ T cells. CD3+ live single cells, CD4+CD3+ live single cells and CD8+CD3+ live single cells were analysed separately for CFDA-SE dye dilutions. Isotype controls and FMOs were used as experimental and gating controls. Proliferating cells are gated using unstimulated controls. Results were presented as % of proliferating cells using the above gating strategy.
