# Supplementary material for: Promoting Immune Response of Human Vascular Endothelial Cells by Bevacizumab: Insights into the Immune Supportive Role of Anti-VEGF Therapy
Source: Int J Mol Sci. 2025 Jun 29;26(13):6280. doi: 10.3390/ijms26136280 (PMC12250103; doi:10.3390/ijms26136280)
Supplement: Supplementary file 1 [file ijms-26-06280-s001.zip › Supplementary Videos_Jia et al.pptx]

## Slide 1
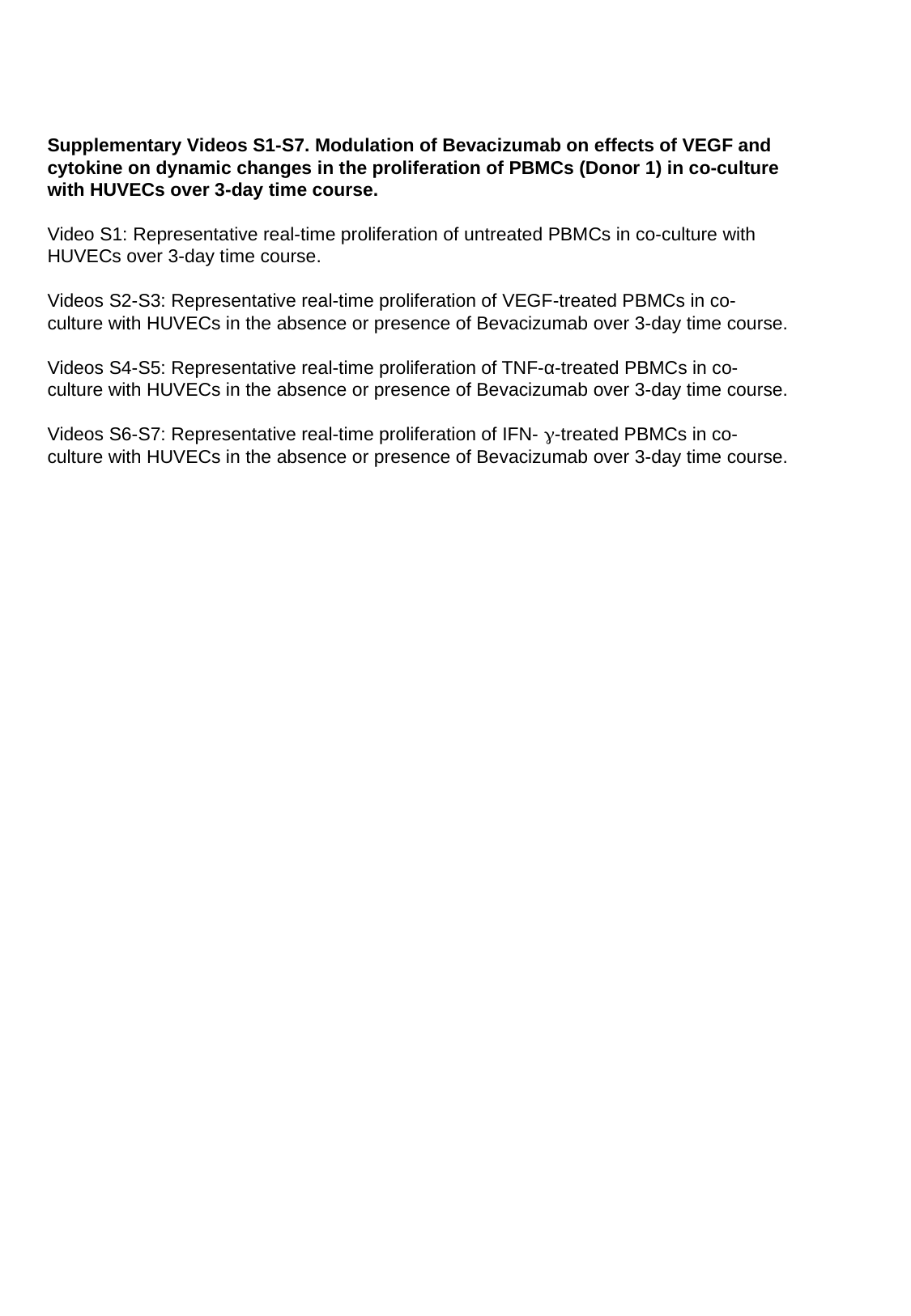

Supplementary Videos S1-S7. Modulation of Bevacizumab on effects of VEGF and cytokine on dynamic changes in the proliferation of PBMCs (Donor 1) in co-culture with HUVECs over 3-day time course.
Video S1: Representative real-time proliferation of untreated PBMCs in co-culture with HUVECs over 3-day time course.
Videos S2-S3: Representative real-time proliferation of VEGF-treated PBMCs in co-culture with HUVECs in the absence or presence of Bevacizumab over 3-day time course.
Videos S4-S5: Representative real-time proliferation of TNF-α-treated PBMCs in co-culture with HUVECs in the absence or presence of Bevacizumab over 3-day time course.
Videos S6-S7: Representative real-time proliferation of IFN- γ-treated PBMCs in co-culture with HUVECs in the absence or presence of Bevacizumab over 3-day time course.
